# Supplementary material for: Adaptive Ketogenic–Mediterranean Protocol (AKMP) in Real Clinical Practice: 14-Week Pre–Post Cohort Study on Glucolipid Markers and Safety
Source: Nutrients. 2025 Nov 14;17(22):3559. doi: 10.3390/nu17223559 (PMC12655798; doi:10.3390/nu17223559)
Supplement: Supplementary file 1 [file nutrients-17-03559-s001.zip › Supplementary File S1 STROBE.pdf]

Supplementary File S1. STROBE

STROBE item	Recommendation	Where reported
1. Title/abstract	Indicate study design in the title/abstract	Title; Abstract (Background/Objectives/Methods/Results)
2. Background	Scientific rationale and justification	Introduction
3. Objectives	Specific objectives and hypotheses	Introduction (last paragraphs)
4. Study design	Key elements of design stated early	§2.1 (Study design and ethics)
5. Setting	Setting, locations, relevant dates	§2.1–§2.3; §3.1 (recruitment/follow-up dates)
6. Participants	Eligibility criteria; sources and methods of selection	§2.2 (Criteria and procedure)
7. Variables	Clearly define outcomes, exposures, confounders, effect modifiers	§2.3 (Intervention); §2.6–§2.8 (indices and composition)
8. Data sources/measurement	Methods of assessment for each variable	§2.4–§2.8 (preanalytical, analytical, BIA)
9. Bias	Efforts to address potential sources of bias	§2.10 (temporal blinding of lab; data management)
10. Study size	How study size was arrived at	§2.1 (convenience sample; no formal calculation)
11. Quantitative variables	How quantitative variables were handled	§2.6–§2.9 (definitions, conversions, rounding policy)
12. Statistical methods	All methods: tests, subgroups, missing data handling	§2.9 (paired tests; sex strata; Δ~Δ correlations; complete-case)

STROBE item	Recommendation	Where reported
13. Participants (flow)	Numbers at each stage; flow diagram recommended	§3.1; Figure 1
14. Descriptive data	Baseline characteristics; missing data numbers	§3.1; Table 1
15. Outcome data	Numbers of outcome events or summary measures over time	§3.2–§3.4; Tables 2A–2C, 3A–3C, 4A–4C, 5A–5C, 6A–6C, 8A–8C; Table 9 (correlations)
16. Main results	Estimates and precision; adjusted analyses	§3.2 (primary, co-primary, secondary; CIs and p)
17. Other analyses	Subgroups, interactions, sensitivity analyses	§3.2 (by sex); §3.3 (pre-specified Δ~Δ)
18. Key results	Summary of key findings with reference to objectives	§4 (Discussion, items 1–7); §5 (Conclusions)
19. Limitations	Limitations with direction and magnitude of potential bias	§4 (Strengths/limitations; ALT/AST not available; no control group)
20. Interpretation	Overall interpretation considering objectives, limitations, multiplicity	§4 (domain-wise synthesis); §5 (Conclusions)
21. Generalizability	External validity	§4 (real-world translatability)
22. Funding/ethics	Funding sources; ethical approvals	§2.1 (ethics); “Funding/Conflicts of Interest”
