# Supplementary material for: Adaptive Ketogenic–Mediterranean Protocol (AKMP) in Real Clinical Practice: 14-Week Pre–Post Cohort Study on Glucolipid Markers and Safety
Source: Nutrients. 2025 Nov 14;17(22):3559. doi: 10.3390/nu17223559 (PMC12655798; doi:10.3390/nu17223559)
Supplement: Supplementary file 1 [file nutrients-17-03559-s001.zip › Supplementary File S2. TREND.pdf]

Supplementary File S2. TREND

TREND domain / Item	Compliance and content	Where reported
Title/abstract	Design (14-week pre–post cohort), population, intervention, outcomes	Title; Abstract
Background	Clinical/scientific rationale	Introduction
Objectives/hypotheses	Improvement in glucose–insulin axis and glycolipid indices; safety	Introduction; §2.9 (outcomes)
Participants	Care setting; inclusion/exclusion; consent	§2.1–§2.2
Interventions	AKMP: macronutrients; Mediterranean matrix; anti-plateau algorithm; schedule	§2.3; “Procedures and schedule”
Unit of analysis	Individuals (adults with overweight/obesity)	§2.2; §3.1
Assignment method	Nonrandomized (pre–post without control)	§2.1
Blinding	Not applicable (diet); lab blinded to time-point	§2.10
Sample size	Convenience sample; no formal calculation	§2.1
Outcomes	Primary (ΔHOMA-IR); gatekeeping co-primary (ΔRC); secondary metabolic/anthropometry/safety	§2.9; §2.6–§2.8; §3.2–§3.4
Outcome measurement	Analytical procedures, BIA, definitions (HOMA-IR, TyG, RC, eGFR)	§2.4–§2.8
Time period	Recruitment 2024; 14-week intervention; windows	§2.1–§2.3; §3.1
Statistics	Paired tests; sex strata; Δ~Δ correlations; Cohen’s d	§2.9
Protocol deviations	Documented in Dietopro®; fidelity monitored	§2.3 (modifications; fidelity)
Participant flow	Assessed, included, completed; STROBE diagram	§3.1; Figure 1
Recruitment	Dates of accrual and follow-up	§2.1–§2.2; §3.1

TREND domain / Item	Compliance and content	Where reported
Baseline data	Demographic/anthropometric features	Table 1
Numbers analyzed	Complete-pairs (complete-case); n per table	§2.9; Tables 2–8; Table 9
Results/estimates	Absolute and relative changes; 95%CIs; p	§3.2–§3.4; Tables 2–8
Ancillary analyses	By sex; pre-specified correlations	§3.2 (by sex); §3.3 (Table 9)
Adverse events	Safety and SAEs (0); safety biomarkers	§3.4 (Safety)
Interpretation/generalizability	Domain-wise discussion; clinical applicability	§4; §5
Ethics/funding	IRB, consent, funding, conflicts	§2.1; “Funding/Conflicts of Interest”
