# Supplementary material for: Adaptive Ketogenic–Mediterranean Protocol (AKMP) in Real Clinical Practice: 14-Week Pre–Post Cohort Study on Glucolipid Markers and Safety
Source: Nutrients. 2025 Nov 14;17(22):3559. doi: 10.3390/nu17223559 (PMC12655798; doi:10.3390/nu17223559)
Supplement: Supplementary file 1 [file nutrients-17-03559-s001.zip › Supplementary File S3. TIDieR.pdf]

Supplementary File S3. TIDieR

1. **Brief name** — *Adaptive Ketogenic–Mediterranean Protocol (AKMP).*
2. **Why (rationale/theory)** — Nutritional ketosis (appetite control; glycolipid improvements) plus Mediterranean matrix (EVOO, oily fish, nuts, non-starchy vegetables), with an anti-plateau algorithm to sustain trunk fat loss; primaries: glucose–insulin axis and remnant cholesterol. (Introduction; §2.3)
3. **What (materials)** — Closed, individualized menus (weighed portions) via Dietopro®; operational rules/substitutions; monitoring supplies (Ketostix®; GlucoMen® areo GK for β-OHB); educational material and telematic support. (§2.3)
4. **What (procedures)** — **Phase 1** (weeks 1–12): ≤20 g CH/d; protein 20–40%TEI; fat 50–70%TEI; target β-OHB ≥0.5–0.6 mmol/L. Anti-plateau: if ≥14 days with ketosis, (a) ↑protein 1.2–1.6 g/kg ideal if hunger; or (b) −100–200 kcal/d mainly from fat if satiety adequate, preserving protein. **Phase 2** (weeks 13–14): gradual reintroduction of CH (+50–100 g/d; low glycemic load, high fiber) within Mediterranean matrix. Biweekly clinic (weight, BIA, capillary β-OHB) and twice-weekly remote logs. (§2.3)
5. **Who provided** — Licensed dietitian-nutritionist (7 years clinical experience in ketogenic/Mediterranean diets) with accredited clinical laboratory support. (§2.3; §2.5)
6. **How (delivery mode)** — Individual in-person visits (30–60 min) every 2 weeks + secure messaging for monitoring/barrier resolution. (§2.3)
7. **Where** — Private health center with Dietetics/Nutrition and Clinical Analysis units (Valencia, Spain). (§2.1–§2.2)
8. **When and how much** — 14 weeks total (~8 planned sessions); capillary β-OHB every 15 days; Ketostix® provided at each visit. (§2.3)
9. **Tailoring** — Personalization of menus (preferences, tolerances, target weight); individual energy-protein adjustments per weight trajectory, satiety, feasibility. (§2.3)
10. **Modifications** — Any change in energy/macros/meals recorded in Dietopro® with traceability. (§2.3)
11. **Planned fidelity** — Twice-weekly weight/urine ketones (remote); biweekly capillary β-OHB; systematic adherence review; supplies provided. (§2.3; "Fidelity")
12. **Actual fidelity** — 105/112 completed (7 withdrawals for non-adherence); adherence verified through logs and in-clinic determinations. (§3.1)
