# Supplementary material for: Adaptive Ketogenic–Mediterranean Protocol (AKMP) in Real Clinical Practice: 14-Week Pre–Post Cohort Study on Glucolipid Markers and Safety
Source: Nutrients. 2025 Nov 14;17(22):3559. doi: 10.3390/nu17223559 (PMC12655798; doi:10.3390/nu17223559)
Supplement: Supplementary file 1 [file nutrients-17-03559-s001.zip › Supplementary File S4. Internal Quality Control (IQC) Program.pdf]

Supplementary File S4. Internal Quality Control (IQC) Program

S4.0. Scope and platforms

- **Serum biochemistry panel:** performed in the study laboratory; analyzer **BS-200E (Mindray)** with **Spinreact** and **BioSystems** reagents.
- **Hormonal panel (TSH, FT3, FT4, insulin, cortisol):** performed at **Laboratorio Juan Bta. Montoro, S.L.**, accredited **ISO 15189** and certified **ISO 9001**, using **ADVIA Centaur® XP/XPT (Siemens Healthineers)**.
- The following are presented: (i) **internal controls** from the study laboratory (S4.2), (ii) **internal verification** at Montoro (S4.3), and (iii) **manufacturer's declared precision (CV%)** from the package inserts (S4.4 and S4.5).

S4.1. Control materials and calibrators (according to IFU provided)

Spinreact (Glucose, Total cholesterol, HDL-c D, Urea, ALP, Magnesium, Total bilirubin, CRP-turbilatex, Uric acid)

- Controls: *SPINTROL H Normal and Pathological*.
- Calibrators: GLUCOSE CAL, CHOLESTEROL CAL, HDLc/LDLc CAL, UREA CAL, MAGNESIUM CAL, Bilirubin CAL, CRP-CAL, URIC ACID CAL.

BioSystems (Triglycerides, γ-GT)

- Controls: *Biochemistry Control Serums* (Levels I and II).
- Calibrators: Triglycerides standard (glycerol equivalent to triolein 200 mg/dL).

Siemens (hormones, ADVIA Centaur XP/XPT)

- Recommended internal controls: Siemens controls, two levels, at least once daily and after each calibration or reagent lot change, as specified in the IFU.

S4.2. Serum biochemistry panel — Internal controls of the study laboratory (BS-200E)

Table S4-A. Repeatability (intra-series) and intermediate precision (inter-series)
(n = replicates; values and units as in the control files.)

Analyte	n Inter	Mean Inter (SD)	CV% Inter	n Intra	Mean Intra (SD)	CV% Intra
Albumin	15	3.58 (0.07)	2.01	15	4.27 (0.04)	0.91
Total bilirubin	15	0.90 (0.07)	7.51	15	0.44 (0.05)	11.76
Creatinine	15	1.22 (0.07)	5.55	15	1.49 (0.04)	2.56
Total cholesterol	15	95.27 (1.71)	1.79	15	185.60 (7.49)	4.04
HDL-cholesterol	15	34.60 (4.72)	13.64	15	79.13 (2.03)	2.57
Urea	15	37.20 (1.61)	4.33	15	34.20 (0.86)	2.52
Uric acid	15	4.56 (0.15)	3.19	15	7.37 (0.08)	1.08
Triglycerides	15	115.60 (1.72)	1.49	15	80.53 (1.60)	1.98
CRP (standard, not hs-CRP)	15	7.25 (0.25)	3.41	15	0.69 (0.16)	22.78
Alkaline phosphatase	15	190.20 (10.67)	5.61	15	198.13 (9.07)	4.58
Glucose	15	108.20 (2.62)	2.43	15	96.47 (1.81)	1.87
Magnesium	15	19.01 (1.20)	6.30	15	20.10 (0.49)	2.44
γ-GT	15	36.80 (0.86)	2.34	15	12.87 (0.35)	2.73

S4.3. Hormonal panel — Internal controls at Montoro Laboratory (ADVIA Centaur XP)

Source: Excel "control de calidad interserie/intraserie e intervalo suero control.xlsx".
Calculation: Mean, sample SD (n−1) and CV%. n=10 replicates per condition.
Control intervals (IFU): Insulin 44.8–71.6 mU/L; Cortisol 12.1–21.8 µg/dL; TSH 5.08–7.98 mIU/L; FT3 5.67–7.28 pg/mL; FT4 1.76–2.86 ng/dL.

Table S4-H1. Repeatability (intra-series) and intermediate precision (inter-series)

Analyte (units)	Intra — Mean (SD)	CV% Intra	Inter — Mean (SD)	CV% Inter	Control interval
Insulin (mU/L)	66.03 (0.71)	1.07	65.96 (0.79)	1.19	44.8–71.6
Cortisol (µg/dL)	18.38 (0.52)	2.81	18.01 (0.46)	2.54	12.1–21.8
TSH (mIU/L)	5.38 (0.13)	2.45	5.44 (0.26)	4.84	5.08–7.98
FT3 (pg/mL)	6.34 (0.03)	0.54	6.32 (0.18)	2.84	5.67–7.28
FT4 (ng/dL)	2.20 (0.04)	1.90	2.27 (0.08)	3.48	1.76–2.86

S4.4. Serum biochemistry — Precision declared by the manufacturer (Spinreact, BioSystems IFU)

Table S4-B. Declared repeatability (intra) and reproducibility (inter).

Analyte (method)	Level ~mean	CV Intra	n	CV Inter	n
Glucose-LQ (Spinreact)	98.5 mg/dL	0.59%	20	2.97%	20
	264.8 mg/dL	0.48%	20	2.57%	20
Total cholesterol-LQ (Spinreact)	99 mg/dL	0.84%	20	1.82%	20
	201 mg/dL	0.70%	20	3.26%	20
HDL-c D (Spinreact)	28.0 mg/dL	0.89%	20	4.60%	20
	76.1 mg/dL	1.06%	20	2.71%	20
Triglycerides (BioSystems)	100 mg/dL	1.7%	20	2.6%	25
	245 mg/dL	0.7%	20	1.2%	25
γ-GT (BioSystems)	31 U/L	1.6%	20	4.8%	25
	99 U/L	0.5%	20	1.4%	25

Analyte (method)	Level ~mean	CV Intra	n	CV Inter	n
Alkaline phosphatase (Spinreact)	174 U/L	0.41%	20	3.93%	20
	443 U/L	0.35%	20	2.75%	20
Total bilirubin (Spinreact)	1.53 mg/dL	1.73%	20	1.92%	20
	5.06 mg/dL	1.01%	20	2.18%	20
Urea-LQ (Spinreact)	37.5 mg/dL	2.79%	20	2.65%	20
	120 mg/dL	0.77%	20	1.65%	20
Magnesium (Spinreact)	2.39 mg/dL	1.18%	20	2.99%	20
	4.01 mg/dL	1.73%	20	3.22%	20
CRP-Turbilatex (Spinreact)	8.6 mg/L	6.5%	10	7.7%	10
	16.8 mg/L	3.6%	10	6.6%	10
	50.5 mg/L	1.9%	10	6.3%	10
Uric acid-LQ (Spinreact)	4.46 mg/dL	0.46%	20	—	—
	4.71 mg/dL	—	—	1.37%	20

S4.5. Hormonal panel — Precision declared by the manufacturer (Siemens, ADVIA Centaur XP/XPT IFU)

Table S4-C. Manufacturer's declared precision (CV%) from Siemens IFU

Assay (Siemens)	Level ~mean	CV Intra	CV Total/Inter	n	IFU source
TSH3-Ultra II	0.5–98 mIU/L	1.7–2.5%	2.6–4.0%	80	IFU TSH3-Ultra II
FT3	2.7–14.8 pg/mL	1.3–2.7%	2.0–3.7%	80	IFU FT3
FT4	0.3–6.0 ng/dL	2.2–3.3%	≤8%	80	IFU FT4
Insulin	14–125 mU/L	3.2–4.6%	≤11%	80	IFU Insulin
Cortisol	2.8–50 µg/dL	2.9–4.2%	4.4–6.0%	80	IFU Cortisol
